# Perineal Protrusion Secondary to Imperforate Hymen and Hydrocolpos in an 8-Year-Old Spayed Female Dog

**DOI:** 10.1155/2016/8067967

**Published:** 2016-01-19

**Authors:** Callie L. Blackford Winders, Karen M. Tobias

**Affiliations:** ^1^University of Tennessee College of Veterinary Medicine, 2407 River Drive, Knoxville, TN 37996, USA; ^2^Department of Small Animal Clinical Sciences, University of Tennessee College of Veterinary Medicine, 2407 River Drive, Knoxville, TN 37996, USA

## Abstract

An 8-year-old female spayed Dachshund presented with an acute onset of dysuria, dyschezia, and painful perineal protrusion. A perineal hernia was ruled out on digital rectal examination, and blood work was unremarkable. An extremely dilated vagina was identified on computed tomography, and hydrocolpos was suspected. Endoscopic vaginal exam confirmed the diagnosis of imperforate hymen. The membrane was ruptured digitally and remnants were removed endoscopically, resulting in resolution of clinical signs. Imperforate hymen is a rare congenital abnormality that can result in extensive fluid accumulation within the vagina and subsequent compression of local structures or secondary infection. Treatment of hydrocolpos includes membrane perforation and removal.

## 1. Introduction

Congenital abnormalities of the female canine reproductive tract are uncommonly seen in veterinary practice, with reported occurrence in 0.02–0.05% of the canine population [1]. An imperforate hymen results from failure of the caudal paramesonephric ducts to fuse with the urogenital sinus, leading to an obstruction located between the vagina and the vestibule, just cranial to the opening of the urethra [1, 2]. If vaginal outflow is completely obstructed, accumulation of normal vaginal secretions will result in its distention, which is known as hydrocolpos [2]. Dogs presenting with imperforate hymen often have a history of infertility or clinical signs secondary to vaginal dilation, including dysuria, dyschezia, and abdominal distention [1, 3–5]. This case report describes a case of hydrocolpos secondary to an imperforate hymen resulting in a novel presenting clinical sign: perineal protrusion.

## 2. Case Presentation

An 8-year-old female spayed Dachshund weighing 7.45 kg was presented to the University of Tennessee Veterinary Medical Center with a large, firm, bilaterally symmetrical, painful protrusion in the perineal region that had appeared acutely 2 days before presentation. The dog had a one-month history of dysuria and pollakiuria that was suspected to be a urinary tract infection; when the dog did not respond to amoxicillin-clavulanic acid, a free catch urine sample was submitted for culture. A few colonies of a* Staphylococcus* organism were grown from the culture, and treatment was changed to marbofloxacin. The dog also had a 2-day history of tenesmus associated with the appearance of the perineal protrusion. The dog had been spayed 2 years prior to presentation after multiple types of unsuccessful breeding.

Physical exam abnormalities revealed an otherwise normal dog with an approximately 5 × 4 × 2 cm firm, painful protrusion on midline of her perineal region, ventral to the anus. A fluid-filled structure was noted ventral to the rectum and distal colon on digital rectal exam; no pelvic diaphragm weakness was palpable, making perineal hernia unlikely. Serum chemistry and complete blood count were unremarkable.

**Figure 1:**
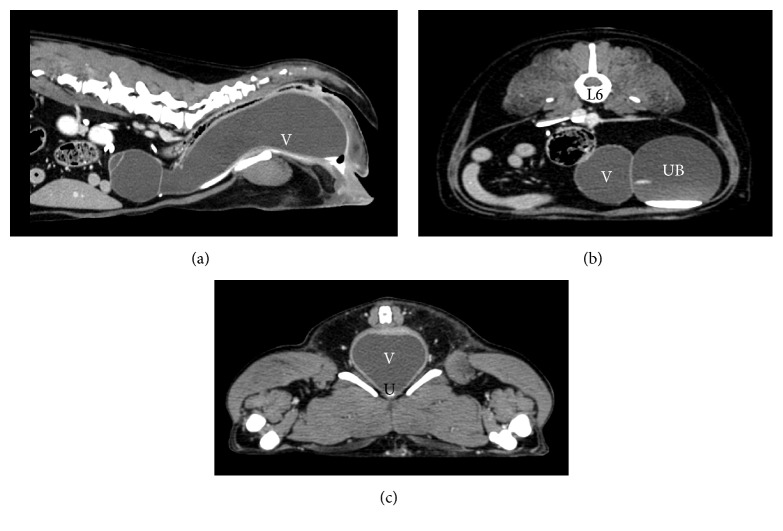
Sagittal (a) and axial (b and c) computed tomography views of the abdomen. (b) and (c) represent the axial view of the abdomen at the level of L6 and the pelvic canal, respectively. The vagina (V), urethra (U), urinary bladder (UB), and L6 are labeled.

The dog was anesthetized for computed tomography (CT) and vaginoscopy. On abdominal and perineal CT (Figures 1(a)–1(c)), a large, tubular, fluid-filled structure was noted, measuring 4 cm in diameter at its widest point and 16.3 cm long. The structure extended from the perineum cranially, blindly terminating at the level of L5. It occupied the majority of the pelvic canal and much of the caudal abdomen, compressing the colon and rectum dorsally and the urethra ventrally and displacing the bladder within the abdomen to the left of midline. There was also mild dilation of both renal pelves and proximal ureters, which could have been secondary to pyelonephritis or backpressure from the dilated urinary bladder. The structure was interpreted to be the vagina and uterine stump and, based on results of physical exam, blood work, and CT, a hydrocolpos secondary to imperforate hymen was suspected. The ratio of vestibulovaginal junction width to maximal vaginal width was 0.825, ruling out vestibulovaginal stenosis.

On digital vaginal exam, a large, thin-walled, fluctuant structure was palpably filling the vaginal lumen just cranial to the urethral meatus. Digital pressure was used against the wall of the structure to perforate it; approximately 200 mL of brown, mucoid fluid was immediately released from the vulva. A sample was collected for cytology and culture. Within a minute, the drainage had ceased, and vaginoscopy was performed, revealing remnants of an imperforate hymen just cranial to the external urethral orifice. No fluid remained in the distended vagina, and no other abnormalities were seen. The remainder of the hymen was removed endoscopically with grasping forceps and submitted for histopathology.

On cytology, the fluid was moderately cellular, primarily consisting of neutrophils with rare clusters of epithelial cells. No bacteria were seen on cytology, and no organisms were cultured from the fluid. Histologically, the tissue was composed of a dense band of fibrous connective tissue covered by a nonkeratinized stratified squamous epithelium. It contained a medium-sized blood vessel and several smaller blood vessels that were surrounded by rare neutrophils. Findings were consistent with the hymen of a nonpregnant mammal. After recovery, the dog's dysuria, pollakiuria, and perineal swelling resolved. The dog was discharged with instructions to complete the previously prescribed course of marbofloxacin. At follow-up 2 months later, the dog was clinically normal.

## 3. Discussion

Hydrocolpos secondary to imperforate hymen has been described in several case reports [3–6]. In the literature, the majority of affected bitches are younger [3, 4]; however, there have been reports of older dogs affected [6]. Dysuria, as seen in the dog of this report, is a common clinical sign of hydrocolpos [1, 3–6]. Urinary tract infection may be initially suspected as the cause of dysuria, as in this dog; however, affected dogs do not respond to antibiotics. Occurrence of a first time urinary tract infection in an older dog or lack of response to appropriate treatment warrants a complete workup because of the high probability of an underlying cause that must be addressed for successful resolution of infection. Recommended diagnostics include a complete blood cell count, serum biochemical profile, urinalysis, urine culture, advanced imaging or endoscopic examination (or both) of the urinary tract, and endocrine testing if suggested by clinical findings and diagnostic results [7]. It was fortunate that this dog had not undergone a “blind stick” for cystocentesis, since it is likely that the hydrocolpos would have been perforated at that time.

Because of the acute appearance of a mass in the perineal region in this older bitch, computed tomographic imaging was performed to rule out neoplasia, perineal herniation with organ entrapment, and other causes of local swelling, such as infection or hemorrhage. In previous reports, hydrocolpos has been diagnosed with survey radiographs and ultrasonography [3–5]. Potential etiologies of hydrocolpos include imperforate hymen, vestibulovaginal stenosis, or an intraluminal mass obstructing fluid outflow of the vagina [1]. Vestibulovaginal stenosis, which is defined by a vestibulovaginal ratio of <0.33, can occur secondary to a variety of developmental vaginal anomalies, including focal vaginal hypoplasia, incomplete perforation of the hymen, vertical vaginal septum, or double vagina, which result in stenosis at the vestibulovaginal junction [8, 9]. In this dog, vestibulovaginal stenosis was ruled out based on measurements taken from the CT. Definitive diagnosis of imperforate hymen is generally made by visualization of the hymen using vaginoscopy or visual speculum examination [3, 5, 6].

Various treatments of imperforate hymen have been described, including fenestration [5]; ovariohysterectomy with removal of the cervix and cranial half of the vagina [3, 4]; and, if vestibulovaginal stenosis is present, resection of the stenotic region followed by vestibulovaginal anastomosis through an episiotomy [10]. All of these techniques have resulted in resolution of clinical signs. However, in one instance of imperforate hymen with secondary pyocolpos that was treated by endoscopic removal of the membrane, the dog developed peritonitis and sepsis after the procedure and died [6]. In cases of infection, it may be more prudent to either resect the infected tissue or at least place drains. In humans, imperforate hymen is treated surgically by making a cruciate incision over the hymen and excising the redundant membrane [11]. Special care should be taken during this process to avoid tearing the vaginal wall. Because of the severe clinical signs associated with hydrocolpos secondary to imperforate hymen, use of a technique that either ablates the tissue or establishes permanent vaginal outflow patency is highly recommended to prevent recurrence of clinical signs. In the presented case, digital perforation and endoscopic removal of membrane remnants were successful in establishing patency and resolving clinical signs.
